# Profiles in conspiracism: Associations with two psychiatric syndromes, religiosity and pandemic-related health behaviors

**DOI:** 10.3389/fpsyt.2022.996582

**Published:** 2022-10-18

**Authors:** Michael J. Minzenberg, Jong H. Yoon

**Affiliations:** ^1^California Neuromodulation Institute, Lafayette, CA, United States; ^2^Department of Psychiatry and Behavioral Sciences, Stanford School of Medicine, Palo Alto, CA, United States

**Keywords:** conspiracy theory beliefs, psychopathology, delusion, religion, social media, pandemic

## Abstract

**Introduction:**

Conspiratorial beliefs are often maladaptive for individuals and dangerous for societies. Other prevalent belief systems such as (normative) religious belief and (pathological) delusional belief show parallels to conspiratorial beliefs, which may also be linked to excessive social media exposure. We conducted an online survey to characterize heterogeneous profiles of conspiracy-mindedness, with respect to these other phenomena.

**Methods:**

Eight hundred and thirty six American adults from online panels completed validated questionnaires including the Conspiracy Mindedness Questionnaire (CMQ), Centrality of Religion Scale (CRS), Peters Delusion Inventory (PDI; 21-item version), and Facebook Addiction Scale (FAS). Additionally, they completed 4 questions addressing categorical belief in the origin of SARS-CoV-2, and pandemic-related health behaviors. Total scores on each questionnaire were *Z-*transformed and entered into K-means cluster analysis. Cluster membership was used in *post-hoc* analyses to compare pandemic-related items.

**Results:**

An optimal solution included 3 clusters with above-mean (high) CMQ and 3 below-mean (low) CMQ scores. The 3 high-CMQ clusters included: (1) high-religion, low-social media addiction; (2) high religion, social media addiction and delusion; (3) low religion and delusion. High-CMQ clusters 1 and 2 each had rates of zoonotic and malevolent viral origin beliefs that were relatively lower and higher than the grand sample rates, respectively. Significant differences in intended pandemic health-related behaviors among the high-CMQ clusters (compared to the rest of the sample) included Cluster 1—high on Precautions and low on Vaccination; Cluster 2—high on Testing. Respondents who endorsed SARS-CoV-2 origin beliefs (across clusters) that were least plausible and most malevolent were least inclined to engage in pandemic health behaviors.

**Conclusions:**

Distinct subpopulations of persons with high conspiracy-mindedness exist, which are highly heterogeneous in their other coexisting beliefs and behaviors. Some of these may be pathological, such as delusional belief and social media addiction-like behavior, and they have varied associations with pandemic-related belief and behavior. These results, while cross-sectional, suggest that the psychological origins and consequences of conspiratorial beliefs may not be unitary. Instead, conspiratorial belief may be a common expression of diverse psychological and social/experiential factors, and in turn exert varied influence on decisions and overt behavior.

## Introduction

Recent world events, including political, large-scale social and global health phenomena (e.g., the COVID-19 pandemic) have turned a spotlight on the widespread expression and influence of conspiratorial beliefs, commonly referred to as conspiracy theories (CT). CT are typically defined in academia as attempts by an individual to explain ultimate causes of significant social and political events with unverifiable claims of secret plots by powerful actors with malevolent intentions (1–3). CT represent organized, elaborated belief structures posited to account for both the origins and consequences related to those large-scale phenomena that are highly salient to the lives of the believers. They can be expressed at a range of social scales, including individuals, local communities and even among politicians who represent large, diverse and dispersed populations. These beliefs have negatively impacted efforts to mitigate a global pandemic and climate change, and even geopolitical stability (4).

One area that remains understudied in the empirical psychology literature of CT is how these beliefs, or the tendency to form them, associate with other elaborated belief systems and practices that are more ubiquitous. In the realm of normative (i.e., non-clinical) populations, religious belief is probably the most common and highly-structured belief system of this kind. CT has been theorized as “quasi-religious representations” with contents, forms and (psychological/social) functions that parallel those found in religious beliefs (5). CT is commonly expressed both within and about religious thought and practice (6), and Wood and Douglas (7) posit specific cognitive mechanisms that religious and conspiratorial thinking both depend on, including pattern detection, agency attribution, compensatory control, and probabilistic reasoning.

Within the spectrum of psychopathology and related phenomena, delusional belief exhibits some similar important basic phenomenological features as CT, as observed in clinical populations characterized by delusional beliefs (which arise from diverse clinical etiologies). These include a general indifference to empirical evidence, a strong and enduring sense of conviction that tends to be unmalleable, and consistent, often predictable effects on the direction of the believer's overt behavior, which can be highly antagonistic. Delusional beliefs can also be detected in a significant portion of the general (i.e., non-clinical) community, 10–15% in many studies, and are distributed in a spectrum in both clinical and non-clinical populations. In addition, cognitive processes play a large role in these non-clinical phenomena [Freeman (8), Clinical Psychology Review], as well as in delusional disorders such as schizophrenia, and include those processes noted by Wood and Douglas (9, 10). Taken together, beliefs that can be characterized as either CT, religious or delusional are found in diverse and geographically-dispersed communities, and generally serve an essential organizing function to integrate and influence one's other beliefs, identity, cognition (e.g., memory content and decision-making) and behavior (both personal and social) (6).

Preliminary associations between CT and either religiosity or delusional ideation has been found in empirical studies. For instance, the degree of CT-proneness has been consistently associated with religiosity (11–14), paranoid or delusional ideation (15–18), and other phenomenological features related to schizophrenia-spectrum conditions (19–25). Importantly, it remains unclear whether either religiosity or delusional/paranoid ideation are distributed evenly among CT adherents. This issue warrants evaluating how they may co-occur in individuals, and whether these patterns may vary, giving rise to heterogeneity among CT adherents. There could exist different ideological “camps,” such as a Religious-CT subpopulation, a Delusional-CT population, and so forth, and important varied social consequences of CT may follow, such as a varied impact of CT on overt behavior (health-related, economic, political). Consequently, varied strategies might be developed to mitigate the personal and societal harm associated with CT-driven behavior.

In addition, while engagement with social media is not generally associated with a discrete, organizing system of belief *per se* (as far as is known), it too is a psychological phenomenon that is strongly implicated in the emergence and spread of CT [(26–33); see discussion in Douglas et al. (2) and Institute for Strategic Dialogue briefings on COVID-19 (4)]. Here too, it remains unclear whether there is important heterogeneity, for instance whether variation in trai*t-*like tendencies to engage with social media predispose individuals to the formation of CT, akin to apparent trai*t-*like tendencies toward conspiratorial thinking that moderate the association of social media exposure with CT (29). From a clinical standpoint, one could reasonably posit that pathological aspects of social media engagement (e.g., addiction-like behavior) serve to amplify CT exposure and may predispose individuals to CT-proneness.

With these considerations in mind, we conducted a survey of US adults to determine how CT-proneness relates to religiosity, delusional belief, and social media addiction-like behavior. We applied a clustering algorithm to establish discrete clusters in the sample, based on profiles of these varied phenomenologies of belief, and social media addiction. We report here a set of profiles that distinguish different subpopulations of individuals with significant “CT-mindedness”, i.e., tendencies to form CT beliefs, and their varied engagement in pandemic health-related behaviors.

## Materials and methods

We conducted an anonymous, panel-based on-line survey integrating data from two different platforms: Lucid (May 12–17, 2021) and Qualtrics (May 12 to June 1, 2021), to diversify the sample source. This study was deemed exempt from Institutional Review Board review (CIRBI, Advarra, Inc.). All respondents were adults located in the United States. Lucid and Qualtrics offer samples that are among the largest available online. Respondents are generally recruited *via* a double op*t-*in procedure, and typically compensated in cash, gift cards or reward points for purchase of consumer items. Basic demographic data is obtained using US Census question phrasing; Race was characterized using categories from the US National Institute of Health. The Lucid sample also obtained self-reported household income, using 24 income brackets ranging from <$14,999 to >$250,000 per year [which we pooled into 4 wider categories to achieve more even sampling]; the Qualtrics sample obtained educational attainment, using categories “high school”; “some college or vocational training,” “college graduate”, and “graduate school or professional degree”). Each platform uses validation checks, e.g., excluding incomplete surveys, excessively “speeded” responses (defined as those with a total completion time < 1/3 of the median time for the full sample), and ensures that each unique IP address can access the survey only once. These platforms generally mitigate the problem of “professionalization” of subjects, and many classic experiments in the empirical social science (including psychology) literature can be readily replicated using these samples (34). This suggests that samples obtained in this manner are suitable for social and health sciences investigation, and are not inordinately constrained or biased relative to samples obtained by other methods. More generally, this approach meets the “fit for purpose” standard that has been promulgated by the American Association for Public Opinion Research [see Coppock and McClellan (34) and Baker et al. (35) for discussion].

All respondents completed the following self-report instruments (each in English). The Conspiracy Mentality Questionnaire [CMQ; Bruder et al. (21)] is a scale measuring trai*t-*like CT-proneness, with 5 items evaluating how likely respondents believe certain general propositions to be true (not pertaining to specific CTs), each on an 11-point Likert scale ranging from 0% “Certainly not” to 100% “Certainly.” It has excellent convergent, discriminant, predictive and cross-cultural validity, and internal and tes*t-*retest reliability (21). Cronbach's alpha in the current sample was 0.861.

The Peters Delusion Inventory [PDI, 21-item version; Peters et al. (36)] queries beliefs that are prevalent in clinical populations characterized by delusional belief (e.g., schizophrenia), (in a Yes/No answer format). For each item with a “yes” response, the instrument branches into 3 subscales (each on a 5-point Likert scale) which measure the degree of Distress, Preoccupation, and Conviction associated with the endorsed belief. It shows good reliability and convergent validity, including in non-clinical samples (36). In the present sample, the PDI-Yes, Distress, Preoccupation and Conviction scales all showed Cronbach's alpha >0.90. The Yes-total was also highly collinear with these other 3 subscales (all bivariate Pearson r > 0.93); therefore, only PDI-Yes total scores were entered in the cluster analysis. The mean PDI-Yes total was 8.4 ± 6.2, reasonably comparable to the mean 6.7 ± 4.4 from the original PDI-21 non-clinical sample (36).

Centrality of Religion Scale [CRS: Huber and Huber (37)] measures the centrality, importance or salience of religious meanings. It is based on 5 theory-defined core dimensions of religiosity: public practice, private practice, religious experience, ideology and intellectual dimensions, conceived of as stable, trai*t-*like features of the individual. Queries of the frequencies of 2 activities (prayer, attendance at religious services) range on a 5-point categorical scale from “never” to “several times a day.” Two items that query more experiential aspects “think about religious issues”, “feeling that God or something divine intervenes…” range on a 6-point categorical scale from “never” to “very often.” A fifth item querying the degree of belief (in “God or something divine”) was on the same 11-point Likert scale as the CMQ items. Because these items were on different scales, they were re-scaled according to a standard, validated method (38). The brief version employed here is worded in a manner that eschews content or associations to specific religions. It shows high construct validity and reliability (37). Cronbach's alpha in the current sample was 0.831.

Facebook Addiction Scale [FAS: Andreassen et al. (39)] is comprised of 6 items that address the psychology (emotions, behaviors) of individuals who are active on Facebook. Items are derived from an explicit correspondence to the six core elements of addiction (salience, mood modification, tolerance, withdrawal, conflict, and relapse). It shows good validity, and internal and tes*t-*retest reliability. Items are rated as frequencies on a 5-point Likert scale, from “very rarely” to “very often.” Cronbach's alpha in the current sample was 0.885.

In addition, we administered 3 items addressing respondents' intention in the current COVID-19 pandemic to (A) take precautions; (B) test for COVID-19 infection, and (C) vaccinate against the coronavirus that causes COVID-19 (SARS-CoV-2). The 3 pandemic items were each scored on a 11-point Likert scale, ranging from “certainly not” to “certainly” (identical to the CMQ Likert response format). We also queried respondents' beliefs about the origin of the coronavirus that causes COVID-19 (SARS-CoV-2), with categorical origin beliefs derived from the most prevalent beliefs at the time of initial study development (April 2020), as determined informally from mainstream media sources (described here: https://allianceforscience.cornell.edu/blog/2020/04/covid-top-10-current-conspiracy-theories/): “It came from a Chinese laboratory dedicated to weapons research”; “It escaped from a non-military Chinese laboratory which had no intent to harm others”; “It had infected other species and then was transmitted to humans”; “It was created or promoted by Bill Gates”; “It does not exist.” The best available evidence at the time of survey supports the account that this virus initially infected other mammalian species, most likely bats (and therefore is a zoonosis), and that the initial infection of humans occurred in the Chinese city of Wuhan, most likely at an open-air market where meat and seafood was sold which was infected with SARS-CoV-2 (40–46). Therefore, the zoonotic account (“other species”) was considered the most accurate belief at the time of study, given the evidence supporting this account, and the general lack of counterevidence available. Each of the other origin beliefs lacked substantive, verifiable evidence from reliable sources at the time of study, and were therefore considered a CT. These were further subcategorized as “non-malevolent” (“escaped from a non-military Chinese laboratory”), as this belief does not attribute malevolent intentions to any agent(s); vs. “malevolent” accounts, each of which imply attributions of malevolent intent to some agent (“weapons research”; “Bill Gates”; “does not exist.”).

Respondents with “fla*t-*score” patterns across items (e.g., consistent scores at the extremes, across all items) were excluded (*n* = 23, or 2.75% of the sample) prior to cluster analysis and inferential testing. *N* = 813 were retained for analysis. The data acquired (total scores for CMQ, PDI-Yes, CRS and FAS) was first transformed to *Z-*scores, and then subject to a K-means cluster analysis.

### Clustering algorithm

In K-Means cluster analysis of a sample, the number of centroids (the imaginary or real location representing the center of the cluster) in the distribution is defined and then every data point is allocated to each cluster by reducing the in-cluster sum of squares (keeping the centroids as small as possible). The K-means algorithm starts with a first group of randomly selected centroids, which are used as the beginning points for every cluster, and then performs iterative calculations to optimize the positions of the centroids (simple Euclidean distance). It halts creating and optimizing clusters when either the centroids have stabilized (i.e., there is no further change in their values because the clustering has been successful), or the defined number of iterations is achieved (default in SPSS set at 10 iterations). The ANOVA results indicate which variables contribute the most the cluster solution, and provide a *post-hoc* verification of significant differences between clusters for each measure (it is not an inferential test *per se*). Larger F values contribute relatively greater separation between clusters. K-means cluster analysis is most valid when measures are on similar scales (e.g., *Z-*scores).

We then stratified all respondents by Cluster membership to conduct *post-hoc* analyses (analysis of variance, *t-*tests, or chi-square, as appropriate) of their inclinations to engage in 3 important health behaviors that aim to mitigate the pandemic effects, and comparative rates of categorical belief in the origin of SARS-CoV-2, the virus that causes COVID-19. All statistical analyses were conducted with SPSS version 27.

## Results

The mean total raw scores (not *Z-*transformed) for each of the 4 major scales is as follows: CMQ, 36.1 ± 10.7, CRS 28.0 ± 12.5, FAS 14.7 ± 6.4, PDI (Yes) 8.4 ± 6.3.

### The *K*-means cluster analysis

**Figure 1:**
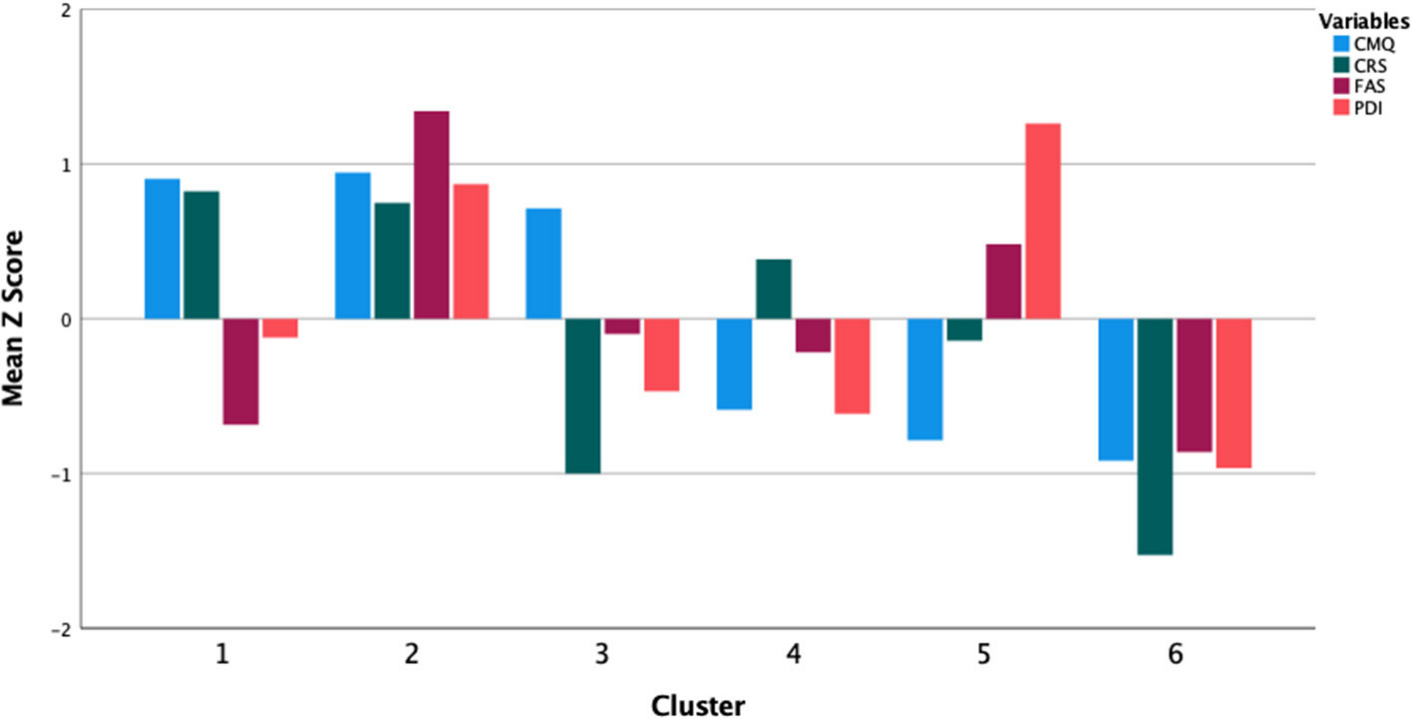
Clusters associating conspiracy-mindedness with religiosity, delusional belief and social media addiction behaviors.

This analysis revealed an optimal 6-cluster solution (e.g., with convergence achieved in <10 iterations, and *post-hoc* analyses of variance of each included measure showing main effects of Cluster with *p*-values < 0.001). Three clusters showed above-mean (high) CMQ scores, and 3 clusters had below-mean (low) CMQ scores (Figure 1). The 3 high-CMQ clusters included: (1) high-religion, low-social media; (2) high-religion, social media and delusion; (3) low religion and delusion. In summary of statistical tests comparing the demographic features of each cluster to the remaining sample (Table 1), the significant demographic differences among the 3 high-CMQ clusters included the following: Cluster 1: older, less educated; Cluster 2: younger, more persons of color, higher income; Cluster 3: more whites, fewer blacks.

**Table 1:** Demographic characteristics of Clusters, with statistical comparisons to the grand sample (Age by *t*-test; others by Chi-square, with *p*-values tabled; ^*^denotes *p* < 0.05).

**Subgroup** **(Cluster)**	**Age (Years)**	**Gender (% F)**	**Race** **(%)**					**Education** **(%)**					**Annual Household Income** **(%)**				
			**White**	**Black**	**Asian/Pacific Islander**	**Native/Indig**	**p**	**HS**	**Some College**	**College Grad**	**Grad/Prof Deg**	**p**	**<15K**	**15-35K**	**35-75K**	**>75K**	**p**
Grand Sample	41.3 ± 18.7	52.6	66.9	21.8	6.8	1.6	N/A	23.3	26.7	34.6	15.4	N/A	29.7	23.9	25.4	21.0	N/A
1	*48.5 ± 19.4	60.1	76.7	18.8	4.5	0.0	0.141	30.9	35.3	20.6	13.2	0.028	35.0	25.0	18.3	21.7	0.543
2	*34.2 ± 13.8	46.0	54.6	31.9	8.4	5.0	<0.001	26.9	19.4	35.8	17.9	0.497	27.5	19.6	13.7	39.2	0.004
3	39.3 ± 16.5	50.9	80.4	10.8	5.9	2.9	0.019	21.6	35.3	35.3	7.8	0.278	22.9	29.2	27.1	20.8	0.663
4	*46.5 ± 19.0	56.8	75.8	18.8	4.3	1.1	0.186	26.2	25.2	37.9	10.7	0.394	33.7	16.9	34.9	14.5	0.029
5	*29.5 ± 12.0	49.3	50.8	35.6	13.6	0.0	<0.001	23.0	18.9	43.2	14.9	0.263	31.7	33.3	22.2	12.7	0.122
6	*48.9 ± 20.8	49.0	83.2	11.6	4.2	1.1	0.027	5.7	30.2	32.1	32.1	<0.001	21.1	21.1	34.2	23.7	0.441

Cluster 2 exhibited scores on all 3 major belief measures that were above the grand mean. Therefore, we considered the possibility that this may represent a consistent response bias (at the group level) across the measures. We examined the test statistics for Levine's test for equality of variances, derived from each pair-wise *t-*test comparison between Cluster 2 and each other cluster on CMQ, CRS, FAS and PDI-Yes total scores, with the assumption that a group pattern of response bias would be manifest as relatively decreased variance in the cluster compared to other clusters. Here, we found that Cluster 2 score variance was not significantly different (e.g., not lower) in any case, except that it was significantly *higher* in the following comparisons: with Cluster 1 for CMQ, FAS and PDI-Yes; with Cluster 3 on PDI-Yes; Cluster 4 on CMQ and PDI-Yes; with Cluster 5 on CRS, FAS and PDI-Yes; and with Cluster 6 on FAS and PDI-Yes. Therefore, we did not detect any evidence for reduced group variance as a measure of response bias in Cluster 2 on the major belief measures. In contrast, this pattern is more consistent with the common observation of increased variance among groups with more extreme scores.

We also found 3 clusters with below-mean CMQ scores. Cluster 4 exhibited a profile with all scores (CMQ, CRS, FAS and PDI) arrayed more closely around the grand sample mean than those of any other cluster. Cluster 5 showed a curious pattern, with the 2nd-lowest CMQ scores, but also the only cluster (of all 6) with PDI scores that were <1 SD above the mean. Cluster 6 showed the lowest scores on each of the 4 measures.

*Post-hoc* analyses of associations of high-CMQ clusters with pandemic-related belief and behavior

We conducted a total of 13 *post-hoc* analyses and therefore used a Bonferroni-corrected *p*-value for statistical significance with *p* < 0.05/13 = 0.00385. Chi-square tests of SARS-CoV-2 origin beliefs showed a significant effect of Cluster (Chi-square = 116.45, df = 20, *p* < 0.001). Among the high-CMQ clusters, the uneven distribution of SARS-CoV-2 origin beliefs included clusters 1 and 2 each having rates of zoonotic and malevolent origin beliefs that were relatively lower and higher, respectively, compared to grand sample rates. Zoonotic origin: Cluster 1, 24.6%; Cluster 2, 33.1%; grand sample, 37.0%. Malevolent origin: Cluster 1, 61.6%; Cluster 2, 46.8%; grand sample, 38.3%. Cluster 3 was more comparable to the grand sample in rates of Zoonotic belief (38.2 vs. 37.0%) and Malevolent CT beliefs (34.5 vs. 37.0%).

We also conducted a one-way analysis of variance of the 3 pandemic health behavior items, with cluster as the independent factor. Each of the 3 pandemic items showed a significant effect of Cluster: Precautions (*F* = 29.24, df = 5, *p* < 0.001); Testing (*F* = 64.36, df = 5, *p* < 0.001), Vaccination (*F* = 212.45, df = 5, *p* < 0.001).

*Post-hoc t-*tests comparing each high-CMQ cluster with the grand sample mean on each of the 3 pandemic-related health behavior items.

In follow-up to the ANOVA of the 3 pandemic health behavior items, we compared the scores of each high-CMQ cluster to the grand sample score to ascertain how each cluster deviated from the full sample on pandemic behaviors (in a manner analogous to rates of origin beliefs expressed relative to the grand sample). We first transformed scores on each pandemic behavior item to *Z-*scores and then conducted *t-*tests to compare each cluster against the grand sample. Among the 3 high-CMQ clusters (and all test statistics relative to the grand sample), Cluster 1 was significantly higher on Precautions (*t* = 3.15, df = 811, *p* = 0.002; Cohen's *d* = 0.29) and lower on Vaccination (*t* = −3.14, df = 811, *p* = 0.002; Cohen's *d* = −0.29). Cluster 2 was significantly higher on Testing (*t* = 4.61, df = 811, *p* < 0.001; Cohen's *d* = 0.45). Two high-CMQ clusters were each higher than the grand sample on Precautions: Cluster 2 (*t* = 2.37, df = 811, *p* = 0.011; Cohen's *d* = 0.23) and Cluster 3 (*t* = 2.28, df = 811, *p* = 0.017; Cohen's *d* = 0.23), but these did not meet the corrected threshold for statistical significance, and all other comparisons had *p* > 0.05. Among all 6 clusters, the descending ranking of intent to Vaccinate was: 6 > 4 > 2 = 3 > 1 > 5 (“>” here indicating *p* < 0.05, pair-wise differences between adjacent group means in two-sample *t-*tests; “=” indicating no pair-wise differences). Across clusters, the intent to Vaccinate ranked by Origin belief was Zoonosis > Civilian Lab > Weapons Lab > Bill Gates = Not Exist.

## Discussion

In this study, we surveyed adults in the United States to derive profiles of conspiracy-mindedness together with the degree of religiosity, delusional ideation, and social media addiction-like behavior. These first 2 are prevalent phenomena of belief that share important phenomenological and cognitive features with conspiratorial beliefs, while social media addiction-like behavior represents a set of putatively pathological behaviors manifest in engagement of an important global media that has been implicated in conspiracy theories. Using an empirical analysis of the “Euclidean space” of these measures, we identified 6 distinct clusters or profiles. Three of these showed a degree of conspiracy-mindedness exceeding the grand sample mean; we conducted *post-hoc* analyses of these 3 clusters, to characterize associations with important COVID pandemic beliefs and behaviors, and respondents' demographic profiles. These clusters can be summarized thus (based on differences from the grand sample mean): Cluster 1: higher Religion, lower Facebook Addiction; origin beliefs—less zoonotic, more malevolent; higher precautions, lower vaccination; older, less educated; Cluster 2: higher Religion, Delusion, and Facebook Addiction; origin beliefs—less zoonotic, more malevolent; higher testing; younger, more persons of color, higher income; Cluster 3: lower Religion, lower Delusion; more white persons, fewer black persons.

These findings provide unique evidence for important features of heterogeneity among groups of individuals who self-report high levels of conspiracy-mindedness (as a trai*t-*like phenomenon, independent of specific CT beliefs). Cluster 1 showed a salient difference with the highest degree of religiosity in the sample, and was relatively older and less-educated as well. These measures have been associated with CT in prior studies (11–14, 27, 32, 47, 48), and this cluster brings together these characteristics to a distinct profile of older, less-educated religious adherents. Both aging and lower education among adults are associated with less cognitive capacity to analytically and critically evaluate information (49, 50), and these individual differences in cognitive “style” have been associated with both CT and religious belief (51, 52). Religion provides an antecedent framework of belief, and CT has been theorized as “quasi-religious representations” with contents, forms and (psychological/social) functions that parallel those found in religious beliefs (5). These two belief systems may also share underlying cognitive mechanisms (7). The co-occurrence of these in Cluster 1 suggests that for these individuals, their antecedent religious belief and practice may stem from a psychological framework that facilitates the ready assimilation of CT.

Cluster 2, in contrast, showed high conspiracy-mindedness together with high scores on all other phenomenological measures: religiosity, delusional belief, and social media addiction. This distinct profile could suggest a profile of “gullibility” (53) where these individuals have an especially-low threshold for forming beliefs in the face of information that may be ambiguous, implausible or lacking in evidence. Alternatively, these individuals may have high levels of parallel engagement in diverse phenomena of belief and practice (religious, delusional, social media engagement) that interact to confer an unusual vulnerability to forming CT (for instance, social media addiction-like behavior that amplifies the ideational effects of misinformation or delusion-proneness; see below). In this context, the relative degree of social media addiction-like behavior in this cluster is noteworthy. The association of CT with use of social media is well-established (26–33), though the precise psychological, social and technological mechanisms by which social media may influence CT formation and spread remains to be elaborated [some recent work aims to delineate the temporal process of emergence of CT in social media: see Dow et al. (28)]. The salient characteristics of this cluster's profile is the highest level of addiction-like behavior in social media use, together with high levels of self-reported religiosity and delusional ideation. Perhaps the poor self-control and affective factors in social media addiction sets up these users for an amplified, under-controlled exposure to misinformation, which could work merely by simple repetition (54) to influence informational preference and belief formation. This is speculative in the context of the present cross-sectional survey, but it suggests avenues for further empirical study.

Cluster 3, the third high conspiracy-mindedness subgroup, interestingly showed quite low levels of religiosity, and a more modest (average) degree of delusional ideation and social media addiction behavior. It is well-established that CTs often target specific major religions, typically a visible religious minority with political, social or economic salience in the community where the CT arises [e.g., Muslims in the US; Jews in various locales; see Robertson et al. (6)]. However, the present measure of religiosity did not address ideational content related to specific religions or practices that are specific for major religions, but rather measured the respondent's self-reported, non-specific belief, experience and practice. Similarly, we used a trai*t-*like measure of CT-proneness, rather than a measure of belief in specific CTs, therefore lacking in content that might target specific religious communities. It would be of interest to consider in future work whether this subpopulation may have CT directed at religious belief itself, and/or directed at religious communities.

In the context of the current global COVID-19 pandemic, we were also interested in whether discernible profiles of conspiracy-mindedness might be associated with varied beliefs and behavior in response to the pandemic.

Across the full sample, we found that those who believed in the Zoonotic origin of SARS-CoV-2 (which has the best currently-available evidence) were most likely to vaccinate, followed by those who believe a non-malevolent origin CT (Chinese civilian lab), and the lowest intent to vaccinate was observed among those who believe one of 3 common malevolent origin CTs. This suggests a general tendency linking the nature of beliefs (plausibility, malevolence) regarding the origins of the pandemic with engagement in the single most important pandemic-related health behavior attainable by individuals [consistent with the findings in Imhoff and Lamberty (55)].

Among clusters, we found a highly heterogeneous pattern of pandemic-related belief and behavior. Cluster 1, the older, less-educated and highly religious subgroup, tended toward malevolent beliefs about the origin of SARS-CoV-2, with greater COVID precautions but less vaccination against SARS-CoV-2. Cluster 2, the high-religion/delusion/social media subgroup, also expressed greater malevolent beliefs about the origin of SARS-CoV-2, but reported a relatively greater inclination to engage in testing, and no significant difference in precautions or vaccination, compared to the average of the full sample. Cluster 3, the low-religion subgroup, did not report a significantly different engagement in precautions, testing or vaccination. This pattern, taken together, suggests that malevolent beliefs about the origin of the virus responsible for the pandemic are especially prevalent within the present sample among those who reported higher levels of religiosity, and less prevalent among those who reported average or lower levels of religiosity. Cluster 1 in particular may exhibit the most maladaptive belief and behavior in the face of the pandemic, especially given that vaccination against SARS-CoV-2 is a very safe, effective and easily obtained treatment to mitigate a prevalent disease with high rates of serious outcomes, including death (56).

The strengths of the study include the diverse sampling method, the novel combination of measures used (with a solid theoretical and empirical rationale), the rigorous analytic approach to profiling respondents, the novelty of the findings, and the associations with important pandemic health behaviors. Limitations include the cross-sectional nature of the sampling, the inability to ascertain the presence of above-threshold clinical psychopathology, nor the content of religious beliefs that may be involved.

## Conclusion

Distinct subpopulations of persons with high conspiracy-mindedness exist, which are highly heterogeneous in their other coexisting beliefs and behaviors. Some of these are pathological, such as delusional belief and social media addiction-like behavior. For other individuals, conspiracy-mindedness is associated with the degree of religious belief and practice. These profiles have varied associations with pandemic-related belief and behavior. While cross-sectional in nature, these results suggest that the psychological origins and consequences of conspiratorial beliefs may not be unitary. Instead, conspiratorial belief may be a multi-determined, syndromal expression of diverse psychological and social/experiential factors, and in turn exert varied influence on decisions and overt behavior.

## Data availability statement

The raw data supporting the conclusions of this article will be made available by the authors, without undue reservation.

## Ethics statement

The studies involving human participants were reviewed and approved by [Center for IRB Intelligence (CIRBI) Platform] CIRBI, Advarra, Inc. Written informed consent for participation was not required for this study in accordance with the national legislation and the institutional requirements.

## Author contributions

MM conceived and designed the study, acquired and analyzed the data, and was primarily responsible for drafting the manuscript. JY contributed to data analysis, writing and editing of the manuscript. Both authors contributed to the article and approved the submitted version.

## Funding

Funding support from the California Neuromodulation Institute.

## Conflict of interest

The authors declare that the research was conducted in the absence of any commercial or financial relationships that could be construed as a potential conflict of interest.

## Publisher's note

All claims expressed in this article are solely those of the authors and do not necessarily represent those of their affiliated organizations, or those of the publisher, the editors and the reviewers. Any product that may be evaluated in this article, or claim that may be made by its manufacturer, is not guaranteed or endorsed by the publisher.
